# Septic pulmonary embolism and subsequent bilateral pneumothorax in patients undergoing chemoradiotherapy for head angiosarcoma: An autopsy case report and literature review

**DOI:** 10.1097/MD.0000000000031755

**Published:** 2022-11-11

**Authors:** Kaoru Ikejiri, Hiroyuki Goto, Miki Usui, Yuichi Nakayama, Kyoko Sugioka, Asami Ito, Kei Suzuki, Yoshifumi Hirokawa, Keiichi Yamanaka, Hiroshi Imai

**Affiliations:** a Emergency and Critical Care Center, Mie University Hospital, Tsu City, Japan; b Department of Dermatology, Mie University Graduate School of Medicine, Tsu City, Japan; c Department of Diagnostic Pathology, Mie University Hospital, Tsu City, Japan; d Department of Infectious Diseases, Mie University Hospital, Tsu City, Japan.

**Keywords:** case report, central venous port, pneumothorax, septic pulmonary embolism

## Abstract

Septic pulmonary embolism (SPE) and subsequent pneumothorax are rare but serious conditions. We report a case of SPE and pneumothorax caused by central venous port (CV port) infection.

**Patient concerns:**

A 73-year-old woman, who underwent chemoradiotherapy for a head angiosarcoma and a CV port placement, presented with general malaise and myalgia.

**Diagnosis:**

A laboratory examination showed high levels of inflammatory markers. Chest computed tomography showed fluid collection around the CV port and multiple ground-glass opacities and nodular shadows in the bilateral lung field. She was admitted with a diagnosis of SPE due to CV port infection. The port was removed, and antibiotic administration was initiated; however, she was intubated because of refractory septic shock. Methicillin-susceptible *Staphylococcus aureus* was detected in the blood and pus around the port site.

**Interventions:**

Her respiratory status did not improve despite recovering from septic shock, and radiologic findings showed a left pneumothorax and exacerbation of SPE on day 9. Her condition was judged ineligible for surgery for pneumothorax, and chest tube thoracostomy was continued.

**Outcomes:**

Air leaks persisted after chest tube thoracostomy, and her respiratory status did not improve despite ventilator management and recruitment maneuvers. Moreover, a right pneumothorax developed on day 19. Her respiratory status gradually worsened, and she died on day 21. Autopsy showed multiple cavitary lesions in the bilateral lungs and emboli containing organization and inflammatory cells that obstructed the pulmonary arterioles.

**Lessons:**

This case indicates that CV port-related infections are infrequent and difficult to diagnose; understanding the clinical features of SPE is important because of its high mortality rate; and pneumothorax secondary to SPE is a rare but serious condition and is difficult to treat during ventilator management.

## 1. Introduction

Septic pulmonary embolism (SPE) is an uncommon type of nonthrombotic pulmonary embolism in which infected thrombi from a primary infectious site embolize to the pulmonary artery, causing infarctions and focal abscesses in the pulmonary vasculature. SPE has been historically associated with infective endocarditis, intravenous drug abuse, oropharyngeal infection, and septic thrombophlebitis (Lemierre syndrome).^[1]^ However, SPE is now observed in immunocompromised patients and in patients with vascular catheters and implantable devices.^[2,3]^

Central venous (CV) ports are widely used in patients who need long-term continuous intravenous therapy, such as chemotherapy, antibiotic treatment, blood transfusion, and parenteral nutrition. Various complications such as infection and thrombosis have been reported because of the implantation of foreign objects in the body. However, only a few reports have documented cases of CV port-related SPE and subsequent pneumothorax.^[2,4,5]^ Herein, we report a case of CV port-related SPE with septic shock, respiratory failure, and subsequent bilateral pneumothorax during chemoradiotherapy for head angiosarcoma.

## 2. Case presentation

**Figure 1.**
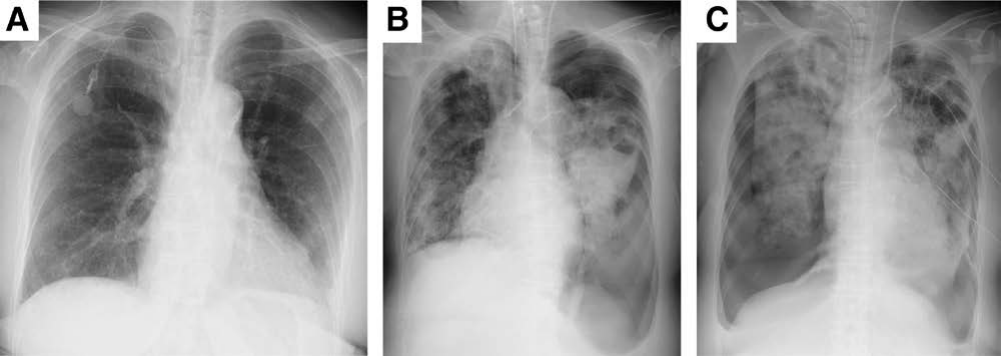
Chest X-ray time course of the case. (A) Recorded before this admission. The CV port was implanted in the right subclavian vein. No obvious lesions were found in the lung field. (B) Recorded on day 9. Left pneumothorax was found. (C) Recorded on day 19. Right pneumothorax was found in addition to existing left pneumothorax. Left pneumothorax persisted even with the insertion of 2 chest tubes. CV = central venous.

**Figure 2.**
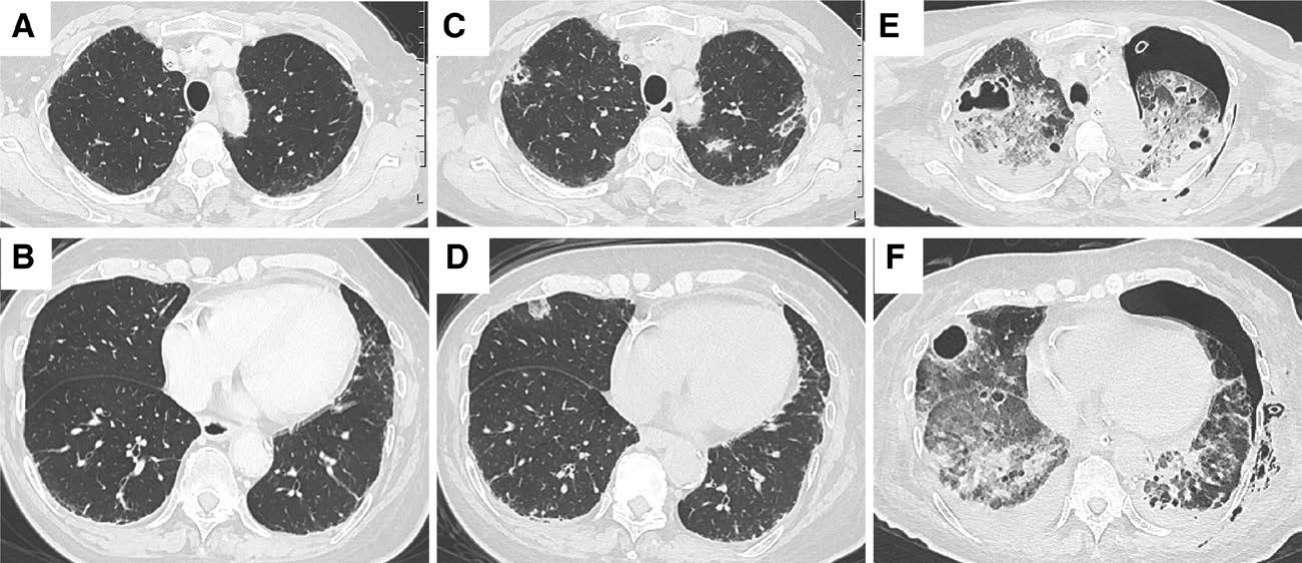
Chest CT time course of the case. (A, B) Recorded before this admission. No remarkable abnormalities were found in bilateral lung field. (C, D) Recorded on day one. Multiple ground-glass opacity and nodular shadow was seen in bilateral lung field. (E, F) Recorded on day 9. Appearance of multiple cavitary lesions and infiltrative shadow in bilateral lung field. CT = computed tomography.

A 73-year-old woman undergoing chemoradiotherapy for head angiosarcoma was admitted to our hospital presenting with general malaise and myalgia. She was diagnosed with head angiosarcoma 7 months prior to admission and was being treated with weekly paclitaxel therapy following radiotherapy. Dexamethasone was administered as premedication. A CV port was implanted in the right subclavian vein 18 days before admission (Fig. 1A), and intravenous fluids were administered through this during the sixth course of treatment. After completion of 6 courses of weekly paclitaxel, the angiosarcoma was reduced, and no remarkable abnormalities were found on computed tomography (CT) (Figs. 2A and B) and in laboratory findings (Table 1). The patient was discharged without any symptoms 3 days prior to admission. The following day, however, general malaise and myalgia appeared and gradually worsened.

**Table 1:** Laboratory findings of the case.

CBC	Day −3	Day 1		Biochemistry	Day −3	Day 1		Blood gas	Day 1	
WBC	6620	6190	/μL	TP	6.0	4.8	g/dL	pH	7.451	mm Hg
RBC	390	388	×10^4^/μL	Alb	3.6	2.2	g/dL	PaCO_2_	34.1	mm Hg
Hb	11.6	11.2	g/dL	BUN	23.4	32.1	mg/dL	PaO_2_	61.1	mmol/L
Ht	35.5	33.8	%	Cr	0.57	0.91	mg/dL	HCO_3_^-^	23.2	mmol/L
Plt	27.1	5.1	×10^4^/μL	Na	143	136	mEq/L	BE	−0.4	mmol/L
				K	4.5	3.9	mEq/L	Lactate	1.7	
**Coagulation**				Cl	106	101	mEq/L			
APTT	28.0	28.0	sec	Ca	9.2	8.2	mg/dL			
PT	10.8	10.4	sec	AST	19	36	IU/L			
PT-INR	0.88	0.89		ALT	32	46	IU/L			
D-dimer		22.2	μg/mL	LDH	207	256	IU/L			
FDP		55.7	μg/mL	ALP	247	420	IU/L			
				T-Bil	0.4	0.8	mg/dL			
				BS	174	232	mg/dL			
				CPK	46	258	IU/L			
				CRP	0.05	32.86	mg/dL			
				PCT		8.67	ng/mL			

Blood gas was taken under 30% administered O_2_.

Alb = albumin, ALP = alkaline phosphatase, ALT = alanine aminotransferase, APTT = activated partial thromboplastin time, AST = aspartate transaminase, BE = base excess, BS = blood sugar, BUN = blood urea nitrogen, Ca = calcium, CBC = complete blood counts, Cl = chloride, CPK = creatine phosphokinase, Cr = creatinine, CRP = C-reactive protein, FDP = fibrin degradation product, Hb = hemoglobin, Hct = hematocrit, INR = international normalized ratio, K = potassium, LDH = lactate dehydrogenase, Na = sodium, PCT = procalcitonin, Plt = platelet, PT = prothrombin time, RBC = red blood cell, T-Bil = total bilirubin, TP = total protein, WBC = white blood cell.

On admission, her initial body temperature, pulse rate, blood pressure, respiratory rate, SpO_2_ and quick sequential organ failure assessment score were 37.1°C, 133 beats/min, 84/53 mm Hg, 33 breaths/min, 92% (ambient air), and 2 respectively. Physical examination revealed generalized myalgia with the strongest point around the right shoulder. A laboratory examination showed thrombocytopenia and an elevated fibrin degradation product and D-dimer level, in addition to high levels of inflammatory markers, compared to 3 days prior (Table 1); sequential organ failure assessment score was 6. Chest CT showed fluid collection around the CV port and multiple ground-glass opacities and nodular shadows in the bilateral lung field (Figs. 2C and D). She was diagnosed with CV port-related sepsis and suspected SPE. After removal of the CV port, pus was observed around the port insertion site. The antibiotic piperacillin-tazobactam plus vancomycin was administered after obtaining blood and pus cultures.

On day 2, she was transferred to the intensive care unit (ICU), because of refractory shock despite receiving an adequate dose of crystalloids and high-dose of vasopressors (noradrenaline up to 0.2 μg/(kg·min) plus vasopressin up to 2 units/h). She was intubated and treated with corticosteroid and renal replacement therapy according to the global sepsis guidelines, in addition to fluid resuscitation and high-dose vasopressors. Because blood and pus cultures yielded methicillin-susceptible *Staphylococcus aureus* on day 3, antibiotics were de-escalated to ampicillin-sulbactam. Repeated transthoracic echocardiogram did not reveal any valvular vegetation or abnormalities. Her hemodynamic status gradually stabilized, and vasopressin as well as renal replacement therapy were discontinued on days 4 and 7, respectively. However, more time was required to taper and discontinue noradrenaline (discontinued on day 10). Repeated blood cultures showed negative results after a week of antimicrobial therapy but showed persistent high levels of inflammatory markers. Moreover, hypoxemic respiratory failure persisted despite adequate positive end-expiratory pressure (PEEP) and a low tidal volume.

On day 9, her oxygen saturation suddenly decreased to 80% after postural change. Chest radiography revealed a left pneumothorax (Fig. 1B), and chest tube thoracostomy was performed. Her oxygen saturation gradually improved after the procedure, but the air leak persisted despite the insertion of 2 chest tubes. Chest CT showed multiple cavitary lesions and infiltrative shadows in the bilateral lung fields, and exacerbation of the ground-glass opacity and nodular shadow, in addition to the left pneumothorax (Figs. 2E and F). Surgical procedures, such as video-assisted thoracostomy for left pneumothorax, were not indicated due to unstable respiratory and hemodynamic status; therefore, treatment with chest tube thoracostomy was continued.

**Figure 3.**
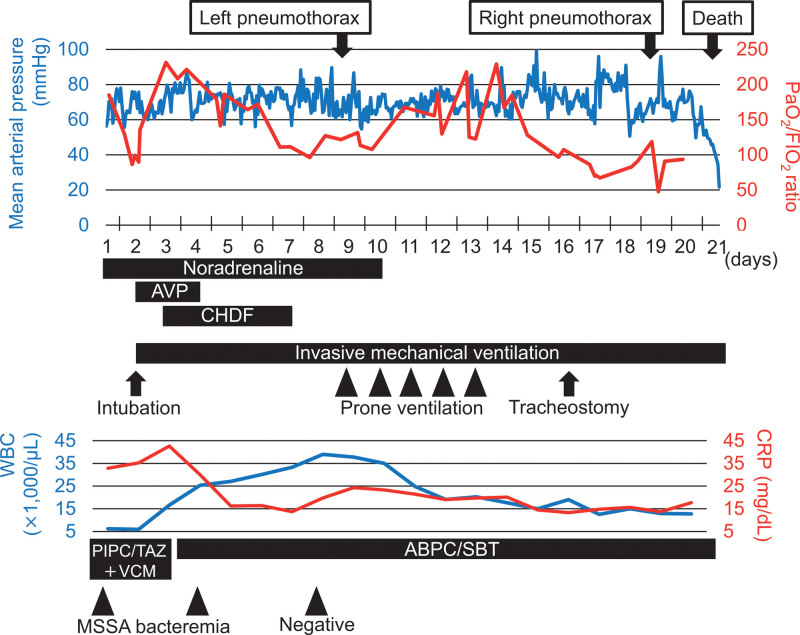
Clinical course of the case. ABPC/SBT = ampicillin-sulbactam, AVP = vasopressin, CHDF = continuous hemodiafiltration, CRP = C-reactive protein, FIO_2_ = fraction of inspired oxygen, MSSA = methicillin-susceptible *Staphylococcus aureus*, PaO_2_ = partial pressure of arterial oxygen, PIPC/TAZ = piperacillin-tazobactam, VCM = vancomycin, WBC = white blood cell.

The etiology of her respiratory failure was suspected to be SPE followed by acute respiratory distress syndrome. In addition to adequate PEEP and low tidal volume setting, prone ventilation was performed to improve oxygenation (from days 9 to 13); however, her respiratory status did not improve. Right one-lung ventilation was attempted because the left pneumothorax and air leak from the chest tubes persisted. This procedure was shortly stopped due to severe hypercapnia. On day 19, her oxygen saturation suddenly decreased to 80% after postural change. Chest radiology showed a right pneumothorax (Fig. 1C), and chest tube thoracostomy was performed. Her oxygen saturation transiently improved after the procedure, but air leakage from all 3 chest tubes persisted. Her respiratory status gradually worsened, and she died on day 21. The clinical course of the patient is shown in Figure 3.

**Figure 4.**
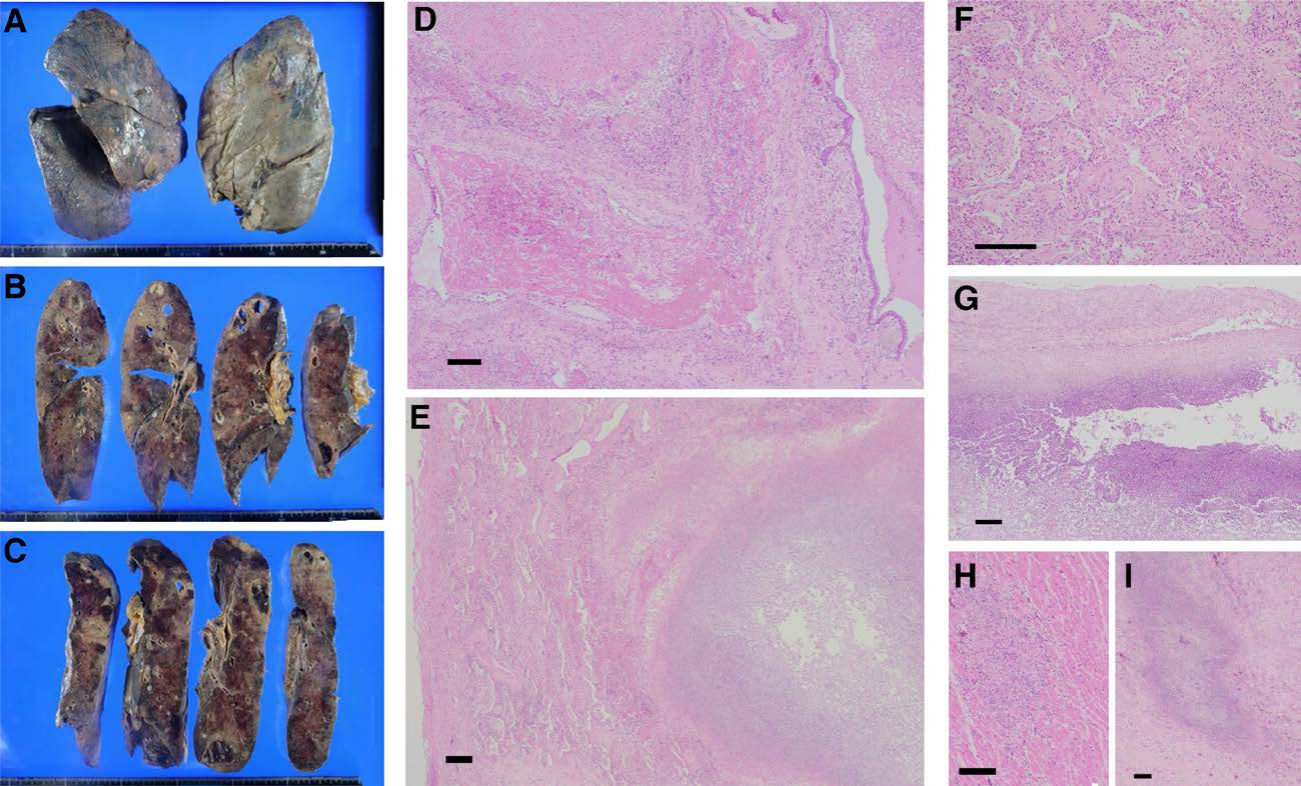
Macroscopic and microscopic findings at autopsy. (A–C) Multiple macroscopical nodular and cavitary lesions were evident in bilateral lung. (D) Emboli containing organization and inflammatory cells obstructed the pulmonary arterioles. (E and F) Normal alveolar architecture was largely destroyed because of interstitial infiltration by fibroblasts. Neutrophil infiltration and abscess were seen in (E) lung, (G) subpleural, (H) ventricular wall, and (I) adrenal medulla. Scale bars in D, F, G, H: 500 µm, and in E, I: 1 mm.

A post-autopsy CT showed bilateral pneumothorax, marked cavitary lesions, and infiltrative shadows in the bilateral lung fields. Moreover, a yellowish fluid at the CV port insertion site was observed, with microscopic neutrophil infiltration in the subcutaneous fatty tissue. Macroscopic findings showed multiple nodular and cavitary lesions in both lungs (Figs. 4A–C). Histological examination revealed emboli containing organized and inflammatory cells that obstructed the pulmonary arterioles (Fig. 4D), in addition to the obliteration of normal alveolar architecture and interstitial infiltration by fibroblasts (Figs. 4E and F). Abscess formation was found in the ventricular wall and renal medulla, in addition to the lungs and subpleura (Figs. 4E and G–I), although findings suggesting cardiac valve vegetation, destruction, and infectious endocarditis were not observed. The findings of systemic metastasis from head angiosarcoma are not shown. These findings and the clinical course confirmed the diagnosis of SPE caused by a CV port-related infection.

## 3. Discussion and conclusion

Here, we report a case of septic shock and respiratory failure due to CV port-related SPE and subsequent bilateral pneumothorax. Although CV ports are widely used, only a few reports have documented cases of CV port-related SPE and subsequent pneumothorax. We found 5 published cases of CV port-related SPE, including this case (Table 2).^[2,4,5]^ The mean age of the patients in these cases was 46.5 years (range, 32–73 years). To the best of our knowledge, this is the first reported case of CV port-related SPE with septic shock, respiratory failure, and delayed bilateral pneumothorax. This case provides 3 important clinical insights.

**Table 2:** Case reports of CV port-related SPE.

	Age/sex	Underlying disease	Time from CV port implantation to SPE onset	Causative pathogen	Definitive antibiotics	Pneumothorax	Treatment of pneumothorax	Outcome	Reference
1	38, F	Breast cancer	4 mo	MRSE	VCM	No		Cured	^[2]^
2	38, F	Breast cancer	4 mo	MSSE	ABPC/SBT	No		Cured	^[2]^
3	32, F	Carotid body paraganglioma	>18 mo	MSSA	CEZ	No		Cured	^[4]^
4	43, M	Stomach cancer	2 mo	Negative	AMPC/CVA	Right	VATS	Cured	^[5]^
5	73, F	Head angiosarcoma	3 wk	MSSA	ABPC/SBT	Bilateral	Chest tube thoracostomy	Dead	This case

The patient in reference^[2]^ suffered twice from SPE. The first episode was in Case 1, and the second episode was in Case 2.

ABPC/SBT = ampicillin-sulbactam, AMPC/CVA = amoxicillin-clavulanate, CEZ = cefazoline, CV port = central venous port, MRSE = methicillin-resistant *Staphylococcus epidermidis*, MSSA = methicillin-susceptible *Staphylococcus aureus*, MSSE = methicillin-susceptible *Staphylococcus epidermidis*, SPE = septic pulmonary embolism, VATS = video-assisted thoracostomy, VCM = vancomycin.

First, CV port-related infections are infrequent and difficult to diagnose. In the present case, the cause of the infection was most likely CV port-related bacteremia spreading from the port pocket infection since pus was present in the pocket after the port was removed. Central line-associated bloodstream infections (CLABSIs) are a major cause of morbidity and mortality worldwide. Approximately 90% of CLABSIs in the United States occur with central venous catheters,^[6]^ and about half of CLABSIs occur in the ICU.^[7]^ A Japanese statistical report showed that the incidence of CLABSI in ICU and general wards was 1.6 and 1.8 per 1000 days of use, respectively.^[8]^ However, the incidence of CV port-related bacteremia and CV port pocket infection was much lower than that of CLABSI (0.016–0.05 and 0.01–0.05 per 1000 days of use, respectively).^[9,10]^ Based on this, we assumed that the possibility of CV port-related infections is likely to be underestimated in patients admitted to general wards. When a patient with an implanted CV port develops an infection, and there is no obvious source of infection on imaging, clinicians should suspect a CV port-related infection and investigate closely.

Second, understanding the clinical features of SPE is important owing to its high mortality rate. As mentioned above, immunodeficiency status and implantable devices have been gaining attention as a risk factor for SPE in recent years.^[2,3]^ The risk factors for SPE in the present case are presumed to be as follows: immunosuppression due to chemotherapy for head angiosarcoma, immunosuppression due to steroids administered regularly as premedication, and less likely, implantable CV port. The clinical manifestations of SPE are nonspecific: fever (85.7%), chest pain (48.81%), dyspnea (48.21%), and cough (41.07%).^[1]^ Therefore, it is difficult to differentiate SPE from other diseases based only on clinical symptoms. Radiologic findings, especially CT findings, are useful in the differential diagnosis of SPE, with multiple nodular opacities (66.42%) being the most commonly observed radiographic signs, followed by cavitary lesions (55.97%), local infiltrations (35.82%), pleural effusions (29.85%), feeding vessel signs (27.61%), and peripheral wedge-shaped lesions (17.16%).^[1]^ CT findings in the present case showed multiple nodular and cavitary lesions; it was relatively easy to include SPE in the differential diagnosis. The mortality rate of SPE is relatively high; 1 systematic review showed that the mortality rate was 10.12%, and septic shock was the most common cause of death.^[1]^ Age, hypotension, and ineffective/delay of empirical antimicrobial therapy were found to be independent risk factors for in-hospital mortality.^[11]^ The patient in the present case recovered from the initial septic shock but died of pneumothorax and subsequent respiratory failure, the etiology of which will be discussed later. SPE should be suspected in patients with a history of underlying disease if they have fever, dyspnea, chest pain, and other clinical manifestations, along with chest CT suggesting multiple nodules or plaques with or without pleural effusion.

Third, pneumothorax secondary to SPE is a rare but serious condition and is difficult to treat when pneumothorax develops during ventilator management. Secondary pneumothorax is a lesser-known complication of SPE; the prevalence of secondary pneumothorax is low (3.4%), but in-hospital mortality is significantly high (28.2%).^[12]^ The possible mechanisms of pneumothorax due to cavitation of nodular lesions include aseptic necrosis resulting from impaired blood flow due to embolization, secondary infection caused by bacterial embolization, and enlarged airspace formed by a check valve mechanism. Several articles have reported cases of SPE and secondary pneumothorax, with infectious endocarditis being the most common cause.^[3,13]^ The cases of CV port-related SPE or subsequent pneumothorax are shown in Table 2. Although 4 of the 5 cases in Table 2 were positive for gram-positive cocci in the blood culture, no obvious findings of valvular vegetations or abnormalities were observed in all cases. The major difference between the patients with SPE-induced pneumothorax reported previously and the patient reported in the present case is that the patient was intubated and mechanically ventilated. Mechanical ventilation is the most common cause of pneumothorax in the ICU setting,^[14]^ and it is widely known that lowering the tidal volume and/or PEEP is necessary to prevent barotrauma. However, it is rare to observe normal lung function during intubation in patients with breathing problems. In this case, the lung parenchyma is fragile due to pulmonary lesions associated with SPE and may be more prone to developing alveolopleural fistula and pneumothorax at lower pressures than during normal respiratory management. After developing a pneumothorax, the management strategy for patients with prolonged air leaks while mechanical ventilation is conservative, including drainage of air via one or more chest tubes, limited airway pressure and volume ventilator settings. However, in this case, the following 2 conflicting strategies made ventilator management difficult. The first was the need to lower the peak pressure and PEEP to prevent the exacerbation of pneumothorax and barotrauma. The second was the need to maintain a high PEEP and several recruitment maneuvers to prevent pulmonary collapse due to acute respiratory distress syndrome secondary to SPE. Consequently, constant inspiratory pressure and PEEP were required to maintain the respiratory status. The fragility of lung parenchyma associated with SPE was also a factor that led to persistent air leak and the development of a contralateral pneumothorax.

At the end of this report, we mention the histological findings of this case. To the best of our knowledge, only a few references in the literature mention the histological features of SPE. One study involving 4 patients undergoing transbronchial lung biopsy described 2 histological findings of SPE; suppurative pneumonia: filling of the alveolar cavity with purulent secretion and marked neutrophil infiltration, and organizing pneumonia: widening of alveolar wall, interstitial infiltration by inflammatory cells, and disappearance of the alveolar cavity with replacement by hyperplastic fibrous tissue.^[13]^ Histological findings of this case showed obliteration of normal alveolar architecture and interstitial infiltration by fibroblasts, which were consistent with the findings discussed above. Moreover, we found non-thrombotic emboli containing organized and inflammatory cells obstructing the pulmonary arterioles. These findings will be important in discussing the pathogenesis and clinical course of septic pulmonary embolism.

## Acknowledgements

We thank all colleagues at the Emergency and Critical Care Center, Department of Dermatology, Department of Diagnostic Pathology, and Department of Infectious Diseases in Mie University Hospital for their assistance. We would like to thank Editage (www.editage.jp) for English language editing.

## Author contributions

All authors read and approved the final manuscript.

**Conceptualization:** Kaoru Ikejiri, Hiroyuki Goto.

**Supervision:** Keiichi Yamanaka, Hiroshi Imai.

**Writing – original draft:** Kaoru Ikejiri, Hiroyuki Goto.

**Writing – pathology part:** Miki Usui.

**Writing – review & editing:** Yuichi Nakayama, Kyoko Sugioka, Asami Ito, Kei Suzuki, Yoshifumi Hirokawa.
